# Supplementary material for: Prevalence and predictors of hypoglycemia in older outpatients with type 2 diabetes mellitus
Source: PLoS One. 2024 Aug 29;19(8):e0309618. doi: 10.1371/journal.pone.0309618 (PMC11361436; doi:10.1371/journal.pone.0309618)
Supplement: Attachment — Submitted filename: PONE-D-24-05623_reviewer.pdf [file pone.0309618.s003.pdf]

PLOS ONE

Prevalence and predictors of hypoglycemia in older adult patients with type 2 diabetes mellitus

--Manuscript Draft--

**Manuscript Number:**	PONE-D-24-05623
**Article Type:**	Research Article
**Full Title:**	Prevalence and predictors of hypoglycemia in older adult patients with type 2 diabetes mellitus
**Short Title:**	Hypoglycemia Predictors in Elderly Type Diabetes Patients
**Corresponding Author:**	Ahmad Al-Azayzih<br>Jordan University of Science and Technology<br>Irbid, JORDAN
**Keywords:**	diabetes mellitus; Geriatric, Elderly; Hypoglycemia; hospital admissions; Jordan.
**Abstract:**	Background: Type 2 diabetes mellitus (DM) is a chronic condition characterized by an inadequate control of glucose levels in the bloodstream. The prevalence of type 2 diabetes has been increasing globally, particularly among older adults who are more susceptible to DM-related complications. Elderly individuals with diabetes are at higher risk of developing hypoglycemia and experiencing multiple chronic conditions simultaneously. Hypoglycemia in older patients can have serious consequences such as cognitive changes, falls, heart problems, and even death.<br>Objective: To assess prevalence, and factors associated with hypoglycemia events in geriatric patients with type 2 diabetes mellitus.<br>Methods: A Cross-sectional study was conducted at King Abdullah University Hospital (KAUH) at the outpatient diabetes clinic over 6 months period. Variables such as socio-demographics, biomedical factors, medication details, and comorbidities were obtained using electronic medical records and patients or caregiver interviews. Recruited patients were followed for hospital admissions, emergency department visits, and deaths over three months follow up period using electronic medical records. Logistic regression models were conducted to identify factors associated with hypoglycemia during the period of three months preceding the clinic visit and hospital admissions/ emergency visits during the follow up period.<br>Results: Electronic medical charts of 640 patients who have type 2 diabetes mellitus and age ≥ 60 years were evaluated. The mean age ± SD was 67.19 (± 5.69) years. Hypoglycemia incidents with different severity levels were prevalent in 21.7 % (n=139) of the patients. Insulin administration was significantly associated with more hypoglycemic events compared to other antidiabetic medication. Patients with liver disease had a significantly higher risk of hypoglycemia, with odds 7.43 times higher than patients without liver disease. Patients with dyslipidemia also had a higher risk of hypoglycemia (odd ratio=1.87). Regression analysis revealed that hypoglycemia and educational level were significant predictors for hospital admission and emergency department (ER) visits. Hypoglycemia was a positive predictor, meaning it increased the odds of these outcomes, while having a college degree or higher decreased the odds of hospital admission and ER visits.<br>Conclusion: Healthcare providers should closely monitor patients on multiple antidiabetic medications, especially those with liver disease and dyslipidemia to prevent occurrence of hypoglycemia events. Educational programs should emphasize the importance of regular blood glucose monitoring to avoid consequent hospital admissions and ER visits related to severe hypoglycemia.
**Order of Authors:**	Ahmad Al-Azayzih
	Roaa J Kanaan
	Shoroq M Altawalbeh
	Karem Alzoubi
	Zelal Kharaba
	Anan Jarab

Powered by Editorial Manager® and ProduXion Manager® from Aries Systems Corporation

Additional Information:	
**Question**	**Response**
**Financial Disclosure**<br><br>Enter a financial disclosure statement that describes the sources of funding for the work included in this submission. Review the submission guidelines for detailed requirements. View published research articles from *PLOS ONE* for specific examples.<br><br>This statement is required for submission and **will appear in the published article** if the submission is accepted. Please make sure it is accurate.<br><br>**Funded studies**<br>Enter a statement with the following details:<br>• Initials of the authors who received each award<br>• Grant numbers awarded to each author<br>• The full name of each funder<br>• URL of each funder website<br>• Did the sponsors or funders play any role in the study design, data collection and analysis, decision to publish, or preparation of the manuscript?<br><br>Did you receive funding for this work?	The author(s) received no specific funding for this work.
**Competing Interests**<br><br>Use the instructions below to enter a competing interest statement for this submission. On behalf of all authors, disclose any competing interests that could be perceived to bias this work—acknowledging all financial support and any other relevant financial or non-financial competing interests.<br><br>This statement is required for submission and **will appear in the published article** if the submission is accepted. Please make sure it is accurate and that any funding sources listed in your Funding Information	The authors have declared that no competing interests exist.

later in the submission form are also declared in your Financial Disclosure statement.<br><br>View published research articles from *PLOS ONE* for specific examples.<br><br>**NO authors have competing interests**<br><br>Enter: *The authors have declared that no competing interests exist.*<br><br>**Authors with competing interests**<br><br>Enter competing interest details beginning with this statement:<br><br>*I have read the journal's policy and the authors of this manuscript have the following competing interests: [insert competing interests here]*<br><br>* typeset	
**Ethics Statement**<br><br>Enter an ethics statement for this submission. This statement is required if the study involved:<br><br>• Human participants<br>• Human specimens or tissue<br>• Vertebrate animals or cephalopods<br>• Vertebrate embryos or tissues<br>• Field research<br><br>Write "N/A" if the submission does not require an ethics statement.<br><br>General guidance is provided below. Consult the submission guidelines for detailed instructions. **Make sure that all information entered here is included in the Methods section of the manuscript.**	Approval for this study was obtained from the Institutional Review Board of KAUH at Jordan University of Science and Technology (Ref. # 531/151/2022). All data were collected after patients completed informed consent forms.

**Format for specific study types**<br><br>**Human Subject Research (involving human participants and/or tissue)**<br>• Give the name of the institutional review board or ethics committee that approved the study<br>• Include the approval number and/or a statement indicating approval of this research<br>• Indicate the form of consent obtained (written/oral) or the reason that consent was not obtained (e.g. the data were analyzed anonymously)<br><br>**Animal Research (involving vertebrate animals, embryos or tissues)**<br>• Provide the name of the Institutional Animal Care and Use Committee (IACUC) or other relevant ethics board that reviewed the study protocol, and indicate whether they approved this research or granted a formal waiver of ethical approval<br>• Include an approval number if one was obtained<br>• If the study involved *non-human primates*, add *additional details* about animal welfare and steps taken to ameliorate suffering<br>• If anesthesia, euthanasia, or any kind of animal sacrifice is part of the study, include briefly which substances and/or methods were applied<br><br>**Field Research**<br><br>Include the following details if this study involves the collection of plant, animal, or other materials from a natural setting:<br>• Field permit number<br>• Name of the institution or relevant body that granted permission	
**Data Availability**<br><br>Authors are required to make all data underlying the findings described fully available, without restriction, and from the time of publication. PLOS allows rare exceptions to address legal and ethical concerns. See the PLOS Data Policy and FAQ for detailed information.	Yes - all data are fully available without restriction

A Data Availability Statement describing where the data can be found is required at submission. Your answers to this question constitute the Data Availability Statement and **will be published in the article**, if accepted.<br><br>**Important:** Stating 'data available on request from the author' is not sufficient. If your data are only available upon request, select 'No' for the first question and explain your exceptional situation in the text box.<br><br>Do the authors confirm that all data underlying the findings described in their manuscript are fully available without restriction?	
**Describe where the data may be found in full sentences. If you are copying our sample text, replace any instances of XXX with the appropriate details.**<br><br>• If the data are **held or will be held in a public repository**, include URLs, accession numbers or DOIs. If this information will only be available after acceptance, indicate this by ticking the box below. For example: *All XXX files are available from the XXX database (accession number(s) XXX, XXX.).*<br>• If the data are all contained **within the manuscript and/or Supporting Information files**, enter the following: *All relevant data are within the manuscript and its Supporting Information files.*<br>• If neither of these applies but you are able to provide **details of access elsewhere**, with or without limitations, please do so. For example:<br><br>*Data cannot be shared publicly because of [XXX]. Data are available from the XXX Institutional Data Access / Ethics Committee (contact via XXX) for researchers who meet the criteria for access to confidential data.*<br><br>*The data underlying the results presented in the study are available from (include the name of the third party*	All relevant data are within the manuscript and its Supporting Information files

and contact information or URL).<br>• This text is appropriate if the data are owned by a third party and authors do not have permission to share the data.<br><br>* typeset	
Additional data availability information:	

Manuscript

Click here to access/download;Manuscript;Hypoglycemia and Elderly Manuscript without tables updated.docx

Prevalence and predictors of hypoglycemia in older adult patients with type 2 diabetes mellitus

Ahmad Al-Azayzih [1,2*], Roaa J. Kanaan [2], Shoroq M. Altawalbeh [2], Karem H. Alzoubi [1,2], Zelal Kharaba [1,3], Anan Jarab [2, 4]

[1] Department of Pharmacy Practice and Pharmacotherapeutics, College of Pharmacy, University of Sharjah, Sharjah 27272, United Arab Emirates

[2] Department of Clinical Pharmacy, Faculty of Pharmacy, Jordan University of Science and Technology. P.O. Box 3030. Irbid 22110, Jordan.

[3] Faculty of Medical Sciences, Newcastle University, Newcastle upon Tyne NE2 4HH, UK

[4] Department of Clinical Pharmacy, College of Pharmacy, Al Ain University, Abu Dhabi Campus, Abu Dhabi 112612, United Arab Emirates

* Corresponding author: Ahmad Al-Azayzih, PharmD, PhD, Associate Professor, Clinical Pharmacy Department, Faculty of Pharmacy-Jordan University of Science and Technology, Irbid, Jordan, 22110. aaazayzih@just.edu.jo. ORCID: 0000-0001-8426-0034

Abstract:

Background: Type 2 diabetes mellitus (DM) is a chronic condition characterized by an inadequate control of glucose levels in the bloodstream. The prevalence of type 2 diabetes has been increasing globally, particularly among older adults who are more susceptible to DM-related complications. Elderly individuals with diabetes are at higher risk of developing hypoglycemia and experiencing multiple chronic conditions simultaneously. Hypoglycemia in older patients can have serious consequences such as cognitive changes, falls, heart problems, and even death.

Objective: To assess prevalence, and factors associated with hypoglycemia events in geriatric patients with type 2 diabetes mellitus.

Methods: A Cross-sectional study was conducted at King Abdullah University Hospital (KAUH) at the outpatient diabetes clinic over 10 months period, from October 1st, 2022 to August 1st, 2023. Variables such as socio-demographics, biomedical factors, medication details, and comorbidities were obtained using electronic medical records and patients or caregiver interviews. Recruited patients were followed for hospital admissions, emergency department visits, and deaths over three months follow up period using electronic medical records. Logistic regression models were conducted to identify factors associated with hypoglycemia during the period of three months preceding the clinic visit and hospital admissions/ emergency visits during the follow up period.

Results: Electronic medical charts of 640 patients who have type 2 diabetes mellitus and age ≥ 60 years were evaluated. The mean age ± SD was 67.19 (± 5.69) years. Hypoglycemia incidents with different severity levels were prevalent in 21.7 % (n=139) of the patients. Insulin administration was significantly associated with more hypoglycemic events compared to other antidiabetic medication. Patients with liver disease had a significantly higher risk of hypoglycemia, with odds 7.43 times higher than patients without liver disease. Patients with dyslipidemia also had a higher risk of hypoglycemia (odd ratio=1.87). Regression analysis revealed that hypoglycemia and educational level were significant predictors for hospital admission and emergency department (ER) visits. Hypoglycemia was a positive predictor, meaning it increased the odds of these outcomes, while having a college degree or higher decreased the odds of hospital admission and ER visits.

Conclusion: Healthcare providers should closely monitor patients on multiple antidiabetic medications, especially those with liver disease and dyslipidemia to prevent occurrence of hypoglycemia events. Educational programs should emphasize the importance of regular blood glucose monitoring to avoid consequent hospital admissions and ER visits related to severe hypoglycemia.

Keywords: Diabetes mellitus; Geriatric, Elderly; Hypoglycemia; Hospital Admissions; Jordan.

Introduction

Diabetes mellitus (DM) is a chronic medical condition characterized by inadequate control of glucose levels in the bloodstream, encompassing a set of metabolic conditions and clinical characteristics marked by elevated blood glucose levels due to impairments in either insulin secretion, insulin function, or both leading to the development of classical symptoms of polydipsia, polyuria, and weight loss for unknown reason [1].

The primary classifications of diabetes are type 1 and type 2 diabetes mellitus. Type 1 diabetes is caused by an autoimmune condition that leads to the loss of pancreatic beta cells function responsible for producing insulin. On the other hand, type 2 diabetes occurs due to a combination of factors, including insulin resistance and a relative lack of insulin release [2]. Insulin resistance is often associated with obesity, a sedentary lifestyle, and advancing age [3, 4]. Genetic factors do play a prominent role in type 1 diabetes development [5], while type 2 diabetes is influenced by various risk factors such as obesity, sedentary and lack of activity lifestyle, unhealthy diet, family history, aging [3, 4].

The prevalence of diabetes has been increasing significantly over the past years. Between 1980 and 2014, the number of people affected by diabetes increased by around four folds from 108 million to 422 million, with the overall prevalence among adults aged 18 and above rising from 4.7% to 8.5% [6]

Elderly individuals, specifically those aged 60 years and above, combat a higher risk of complications associated with diabetes in addition to their aging related syndromes and other chronic comorbidities. Approximately, one-third (around 33%) of individuals in this age group have diabetes and are more susceptible to complications such as hypoglycemia, kidney failure, and heart disease compared to younger individuals with diabetes [7, 8]. As individuals age, the likelihood of experiencing multiple chronic illnesses simultaneously, known as multimorbidity, increases. Multimorbidity negatively affects health-related outcomes and leads to greater healthcare systems utilization and economical expenses while also negatively affecting the quality of life for older adults [9].

The management of multiple chronic conditions in older adults often involves the use of multiple medications, leading to polypharmacy. Polypharmacy refers to the concurrent use of multiple medications, typically defined as five or more. While multiple medications might be necessary for clinical benefits sometimes, polypharmacy can also result in harm or unnecessary medication use. It is associated with negative health outcomes in older adults, including increased risk of death, falls, adverse events, and hypoglycemia, decline in cognitive functions, non-adherence, and hospitalization [10-13].

Hypoglycemia is defined as a drop in blood glucose among patients to below than 70 mg/dl according to American Diabetic Association criteria [14]. Recurrent episodes of hypoglycemia could occur in elderly patients more frequently. Health outcomes reports have identified hypoglycemia as an alarming serious linked to higher rates of emergency department and hospital admission [15].

Older individuals are more prone to experiencing hypoglycemic episodes compared to younger individuals. It is showed that in primary care settings, approximately 11% of participants experienced at least one episode of hypoglycemia within a year, and this rate was higher among older individuals [16]. The incidence of hypoglycemia in care homes is even more significant, with one study indicating a rate as high as 41.9% over a one-year period. This higher incidence can be attributed to the greater presence of comorbidities among the residents in care homes [17].

Existence of hypoglycemia in elderly patients can result in various consequences, such as cognitive changes both in the short and long term, significant falls, cardiac arrhythmia and heart attacks, frailty, and potentially even fatal outcomes [18].

Limited number of studies have addressed the hypoglycemia associated predictors among elderly diabetic patients in Jordan so far. So, this study aims to examine and identify the prevalence of hypoglycemia incidents and predict factors that increase the occurrence of hypoglycemia in elderly patients with type 2 diabetes mellitus.

Methodology:

Study Design and Settings:

A Cross-sectional study was conducted at King Abdullah University Hospital (KAUH) at the outpatient diabetes clinic over 10 months period, from October 1st, 2022 to August 1st, 2023. Patients were eligible if they were 60 years and older, had a confirmed diagnosis of type 2 diabetes mellitus (DM), and were taking at least one diabetic medication. Patients were approached during their scheduled visits to diabetes clinic to evaluate their eligibility and their willingness to participate in the study by an expert clinical pharmacist. Eligible patients were informed about the study goals, process, and voluntary participation. Patients with severe cognitive impairments and hearing problems without caregivers were excluded from the study. Recruited patients were followed for hospital admissions, emergency department visits, and deaths over three months follow up period using electronic medical records.

Patients Characteristics and Data Collection.

Patients characteristics including age, gender, marital status, occupation, monthly income, smoking status, educational level, physical activity family history of DM, and current chronic diseases status in study subjects were obtained from the patients during the interview and from hospital medical charts. Biomedical lab parameters, including fasting blood sugar (FBS), random blood sugar (RBS), glycosylated hemoglobin (HbA1C), low-density lipoprotein (LDL), high-density lipoprotein (HDL), total cholesterol, triglycerides, hemoglobin, systolic blood pressure (SBP), diastolic blood pressure (DBP), as well as medication information including past and present medications, were obtained from the hospital's electronic medical records. Data about hypoglycemia during the three months preceding the clinic visit were obtained from the patients during the interview and validated using medical records and lab results. Hypoglycemia severity levels were assessed based on both American Diabetes Association and The International Hypoglycemia Study Group recommendations [19-21]. Hospital admissions, emergency department visits, and medication refills data were obtained from the electronic records during the three-months period post the interview. The interviews lasted around 10-15 minutes. Charleson comorbidity index was calculated for each patient to indicate morbidity status and to estimate the risk of death from comorbid diseases [22].

Sample size calculation:

A sample size of 385 subjects was required for achieving a 95% confidence interval with a margin of error of 5% [23].

Data analysis:

Data was analyzed using Stata version 17 software (Stata Crop. 2021). Continuous variables such as age, lab results, and number of comorbidities were summarized in terms of their mean and standard deviation. Categorical variables such as gender, body mass index, and marital status were described using frequencies and percentages. Backward stepwise logistic regression models with P value < 0.2 to stay were conducted to evaluate factors associated with hypoglycemia in the period of three months preceding the clinic visit and hospital admission/ emergency visit during the follow up period. A two-sided P value of less than 0.05 was assumed statistically significant.

Ethical Approval and Informed Consent

Approval for this study was obtained from the Institutional Review Board of KAUH at Jordan University of Science and Technology (Ref. # 531/151/2022). All data were collected after patients completed written informed consent forms.

Results

A total of 640 patients with type 2 diabetes aged 60 years and above were recruited in the study, with a mean age ± SD of 67.19 (± 5.69) years. Hypoglycemia was prevalent in 21.7 % of the patients. Of the total subjects, 55.78% were females, 82.81% were married, 84.10% were not living alone, 84.22% were unemployed or retired, and patients 52.34% were with a low monthly income. Only 25.16% of the participants were smokers, and 29.06% were physically active. Additionally, 55.94% of the patients had a family history of diabetes mellitus. A statistically significant difference was identified between the groups with or without hypoglycemia in regard to the family history of diabetes (p=0.003). **(Table 1)**

This study showed that 14.4% of the elderly patients with type 2 diabetes experienced severe level of hypoglycemia (level 3), while 42.4% of the patients experienced moderate level of hypoglycemia (level 2), and 43.2% experienced mild level of hypoglycemic events (level 1). **(Figure 1)**

The results indicated that the mean number of comorbid conditions was 2.72 (SD = 1.47), with hypertension being the most common (79.22%), followed by dyslipidemia (58.13%) and myocardial infarction (16.09%). On average, patients were taking 6.59 (SD = 2.72) medications, including insulin, oral antihyperglycemic agents, and other drugs to treat comorbid conditions. The mean number of medications for diabetes mellitus type 2 was 1.70 (SD = 0.87). The mean value of the Charlson comorbidity index was 3.83 (± 0.91), indicating a moderate severity of comorbid diseases. Among the study sample, 17.97% had hospital admissions during the three months post interview, while emergency department visits were experienced by 8.13% of patients. **(Tables 2 and 3)**

The average blood pressure readings were 136.05 mm Hg (SD = 21.35) for systolic blood pressure and 79.17 mm Hg (SD = 10.51) for diastolic blood pressure. The mean value for HbA1C was 7.70% (SD = 1.68). Average total cholesterol was 6.22 (SD = 26.20) mmole/L while average triglyceride level was 2.76 (SD = 10.47) mmole/L. (**Table 4)**

Our results indicated that the most commonly prescribed antidiabetic medication was metformin followed by sulfonylurea medications (Glimepiride, Gliclazide, Glibenclamide). Metformin was administered in various strengths (500, 850, or 1000 mg) to 501 individuals. Notably, only 107 patients (21.36%) taking metformin experienced hypoglycemia which was not statistically significant association. Furthermore, among the 264 patients receiving sulfonylureas, 54 individuals (20.45%) encountered hypoglycemia, indicating no statistically significant association. However, among the 243 patients prescribed insulin (glargine, detemir, degludec, Aspart, regular, NPH) , a noteworthy 26.75% experienced hypoglycemia, and this association was found to be statistically significant (p=0.016). In the case of DPP4-I (saxagliptin, Linagliptin), with 116 recipients, and GLP1A (liraglutide, simaglutide), involving only 3 patients, the

incidence of hypoglycemia was 26.72% and 33.33%, respectively, although statistical significance was not achieved. Furthermore, SGLT-1, with 40 participants, exhibited a hypoglycemia rate of 25.00%, with no statistically significant association identified. **(Table 5)**

Multivariate logistic regression analysis was applied to examine the potential predictors associated with hypoglycemia. Results showed that patients with a history of liver disease exhibit 7.43 times higher odds of experiencing hypoglycemia compared to those without liver disease (P = 0.020). Furthermore, dyslipidemia as a predictor reveals an odds ratio of 1.87, suggesting an 87% elevated likelihood of hypoglycemia (P = 0.032). Similarly, insulin use has revealed odds of 1.88, meaning that there is 88% increase in the odds of hypoglycemia for individuals receiving insulin (P = 0.015). Moreover, individuals with a family history of diabetes mellitus exhibit 1.82 higher odds of experiencing hypoglycemia episodes compared to those without family history of DM. (P = 0.023). **(Table 6)**

Results of this study showed that individuals who have experienced hypoglycemia had 1.95 times higher odds of hospital admissions and emergency department (ER) visits compared to those without hypoglycemia episodes. (95% C.I = 1.25-3.04, P = 0.003). Also, Patients who had higher educational level exhibit lower odds (OR=0.61) compared to patients who did not complete their education, this mean that hospital admissions and ER visits in patients with higher education were lower by 39% compared to patients with lower education level (95% CI = 0.40-0.94). **(Table 7)**

Discussion

Diabetes Mellitus Type 2 is highly prevalent among population of Jordan and neighbor countries. Our study was conducted to address the prevalence of hypoglycemic events among geriatric patients with type 2 diabetes from Jordan. Furthermore, the study aimed to identify the main predictors linked to development of hypoglycemic conditions among elderly diabetic population.

Results of the current study estimated that prevalence of hypoglycemia among elderly patients was 21.7 %. Hypoglycemia prevalence among type 2 diabetes patients was reported previously in other studies ranged from 12% to 57.44% [24-26]. According to a previous cross-sectional study conducted in the diabetes outpatient clinics, the hypoglycemic events prevalence was higher among patients on insulin and oral hypoglycemic agents compared to those who were on diet alone [24]. Hypoglycemia is generally associated with severe negative consequences including increased risk of cardiac arrhythmias, falls, and even mortality [27, 28]. Our study indicated that 14.4% of the study patients reported severe hypoglycemia, while 42.4 and 43.2% of the patients reported moderate and mild hypoglycemia, respectively. A systematic and meta-analysis population-based study included 46 studies in their analysis had prevalence estimate of

hypoglycemia of 45 % for mild and moderate hypoglycemia, while 6% prevalence for severe hypoglycemia [25]. Our study indicated that 14.4% of the study patients reported severe hypoglycemia.

Our study has evaluated multiple variables as suspected predictors for increased hypoglycemic events among patients. The number of diabetes medications was linked significantly to the hypoglycemia events occurrence. Previous studies have shown a similar association between two or more antidiabetic medications compared to a single medication and increased the risk of hypoglycemia in diabetic patients [29-31].

Glycosylated hemoglobin (HbA1c) is a reliable indicator of long-term control of blood glucose in DM patients. The study has shown that mean level of HbA1c (7.7%) was comparable and elevated in all patients (with and without hypoglycemia). While this indicates poor control of blood glucose among elderly patients in this study, it might to some extent explain the low prevalence of hypoglycemic events in DM patients. Improper antidiabetic selection, low dose or number of antidiabetic medications, and poor medication adherence can lead to poor control of blood glucose expressed through HbA1c and thus, less chance of developing hypoglycemic state. Reports have looked the association between hypoglycemia prevalence and HbA1C level showed before contradicting results, while one study has indicated that prevalence of hypoglycemia increases for HbA1c readings <7.0% [32], the other study showed that increased HbA1c level increased the odds for hypoglycemia related hospitalization [33].

According to the American Diabetic Association (ADA) recommendations for blood pressure (B.P) goal in DM patients, the B.P should below <140/90 mm Hg unless the patients have or at higher risk of cardiovascular events, then B.P should be <130/80 mm Hg [34-36]. The Mean B.P readings for this study patients showed uncontrolled B.P for both groups with hypoglycemia and non-hypoglycemia. While it is crucial to keep the B.P values according to ADA recommendation, mainly through using more than one antihypertensive medication, thusly, more risk of hypoglycemia might be generated based on reports associating antihypertensive medications including β-Blockers use with more risk of hypoglycemia [37]. This risk-benefit balance should be evaluated by prescribers to maximize the health outcomes for patients with cardiovascular problems (e.g., hypertension) and diabetes and minimize risk of hypoglycemia [16].

Cardiovascular diseases, dyslipidemia, hypothyroidism, and chronic kidney disease were the most common chronic conditions encountered in this study. Numerous chronic conditions including chronic kidney diseases, hypothyroidism, and liver diseases were considered as independent risk factors associated with drop of blood glucose and have been linked to elevated hypoglycemic events in diabetic and non-diabetic people [38-40].

The presence of chronic comorbidities including liver conditions and dyslipidemia were linked to an increase in the risk of hypoglycemic events in this study. On the other hand, chronic kidney disease and hypothyroidism assessment failed to establish a significant association with more hypoglycemic instants. Reports explained the association between liver conditions and raised odds of hypoglycemia due to poor glycogen level and malnutritional status among these patients resulted in existence of hypoglycemia [40]. Dyslipidemia and hypertriglyceridemia were clearly linked to insulin resistance and thus, hyperglycemia [41]. Different pharmacological studies have examined the effects of drugs indicated for dyslipidemia on the blood glucose level. These studies concluded that Statins and ezetimibe administration could produce notable hypoglycemia [42, 43]. Hence, the hypoglycemic events in patients with co-existed dyslipidemia may be due to pharmacological effects of drugs used rather than the nature of the dyslipidemia condition itself.

From another perspective, this study has looked at the variables that might be associated with more frequent admission to either emergency department visits or admission to hospital. In current study, Patients who had developed hypoglycemia were more subjected to admission to emergency department and hospital internal medicine department. A study has reported that recurrent emergency department visits after hypoglycemic events were more common in those taking oral antidiabetic agents compared to insulin only [44]. Another study indicated hypoglycemia as a major risk factor for hospital admission among geriatric patients suffering of diabetes [15].

Study limitations.

This study might be subjected to recall bias, even though the study methodology ensured to assess the hypoglycemic events carefully based on both electronic recorded data and patient’s interview.

Conclusion:

Our study indicated a relatively high prevalence of hypoglycemia events occurrence among elderly diabetic patients. Patients with co-existing liver diseases and dyslipidemia were significantly associated with more odds of hypoglycemia events. Educational programs should be implemented to increase awareness of hypoglycemia risk factors in diabetic geriatric population. Also, prescribers should conduct more strict assessment for drug-induced hypoglycemia to avoid unwanted adverse events of hypoglycemia among elderly patients.

Acknowledgment:

The research was approved by the Jordan University of Science and Technology (JUST) Research Counsel and Deanship of Research (Approval /Research number is 559-2022).

Funding:

The authors did not receive any source of funds to conduct this study.

Conflict of Interest:

The authors have no conflict of interest to declare.

References

1. Schuster DP, Duvuuri V. Diabetes mellitus. Clin Podiatr Med Surg. 2002;19(1):79-107. Epub 2002/01/25. doi: 10.1016/S0891-8422(03)00082-X. PubMed PMID: 11806167.
2. Genuth SM, Palmer JP, Nathan DM. Classification and Diagnosis of Diabetes. In: Cowie CC, Casagrande SS, Menke A, Cissell MA, Eberhardt MS, Meigs JB, et al., editors. Diabetes in America. 3rd ed. Bethesda (MD) interest.2018.
3. Roberts CK, Hevener AL, Barnard RJ. Metabolic syndrome and insulin resistance: underlying causes and modification by exercise training. Compr Physiol. 2013;3(1):1-58. Epub 2013/05/31. doi: 10.1002/cphy.c110062. PubMed PMID: 23720280; PubMed Central PMCID: PMCPMC4129661.
4. Cigolle CT, Blaum CS, Lyu C, Ha J, Kabeto M, Zhong J. Associations of Age at Diagnosis and Duration of Diabetes With Morbidity and Mortality Among Older Adults. JAMA Netw Open. 2022;5(9):e2232766. Epub 2022/10/01. doi: 10.1001/jamanetworkopen.2022.32766. PubMed PMID: 36178688; PubMed Central PMCID: PMCPMC9526092 National Institute on Aging (NIA) during the conduct of the study and funding from the Michigan Health Endowment Fund, the Veterans Affairs (VA) Office of Rural Health, and the Patient Safety Center outside the conduct of the study. Mr Kabeto reported receiving grants from the University of Michigan during the conduct of the study. Dr Zhong reported receiving grants from the National Institutes of Health during the conduct of the study. No other disclosures were reported.
5. Steck AK, Rewers MJ. Genetics of type 1 diabetes. Clin Chem. 2011;57(2):176-85. Epub 2011/01/06. doi: 10.1373/clinchem.2010.148221. PubMed PMID: 21205883; PubMed Central PMCID: PMCPMC4874193.
6. (WHO) WHO. Diabetes Fact Sheet.
7. Sloan FA, Bethel MA, Ruiz D, Jr., Shea AM, Feinglos MN. The growing burden of diabetes mellitus in the US elderly population. Arch Intern Med. 2008;168(2):192-9; discussion 9. Epub 2008/01/30. doi: 10.1001/archinternmed.2007.35. PubMed PMID: 18227367.
8. Chentli F, Azzoug S, Mahgoun S. Diabetes mellitus in elderly. Indian J Endocrinol Metab. 2015;19(6):744-52. Epub 2015/12/23. doi: 10.4103/2230-8210.167553. PubMed PMID: 26693423; PubMed Central PMCID: PMCPMC4673801.
9. Kuzuya M. Era of geriatric medical challenges: Multimorbidity among older patients. Geriatr Gerontol Int. 2019;19(8):699-704. Epub 2019/08/10. doi: 10.1111/ggi.13742. PubMed PMID: 31397060.
10. Milton JC, Hill-Smith I, Jackson SH. Prescribing for older people. BMJ. 2008;336(7644):606-9. Epub 2008/03/15. doi: 10.1136/bmj.39503.424653.80. PubMed PMID: 18340075; PubMed Central PMCID: PMCPMC2267940.
11. Caughey GE, Roughead EE, Vitry AI, McDermott RA, Shakib S, Gilbert AL. Comorbidity in the elderly with diabetes: Identification of areas of potential treatment conflicts. Diabetes Res Clin Pract. 2010;87(3):385-93. Epub 2009/11/20. doi: 10.1016/j.diabres.2009.10.019. PubMed PMID: 19923032.
12. Aljeaidi MS, Tan EC. The association between polypharmacy and cognitive ability in older adults: A national cohort study. Res Social Adm Pharm. 2022;18(3):2505-9. Epub 2021/05/15. doi: 10.1016/j.sapharm.2021.04.018. PubMed PMID: 33985890.
13. Al-Musawe L, Martins AP, Raposo JF, Torre C. The association between polypharmacy and adverse health consequences in elderly type 2 diabetes mellitus patients; a systematic review and meta-analysis. Diabetes Res Clin Pract. 2019;155:107804. Epub 2019/08/04. doi: 10.1016/j.diabres.2019.107804. PubMed PMID: 31376400.
14. American Diabetes A. 6. Glycemic Targets: Standards of Medical Care in Diabetes-2021. Diabetes Care. 2021;44(Suppl 1):S73-S84. Epub 2020/12/11. doi: 10.2337/dc21-S006. PubMed PMID: 33298417.
15. Pasciak WE, Berg DN, Cherlin E, Fried T, Lipska KJ. Qualitative analysis of reasons for hospitalization for severe hypoglycemia among older adults with diabetes. BMC Geriatr. 2021;21(1):318.

Epub 2021/05/19. doi: 10.1186/s12877-021-02268-w. PubMed PMID: 34001014; PubMed Central PMCID: PMCPMC8130109.
16. Bramlage P, Gitt AK, Binz C, Krekler M, Deeg E, Tschope D. Oral antidiabetic treatment in type-2 diabetes in the elderly: balancing the need for glucose control and the risk of hypoglycemia. Cardiovasc Diabetol. 2012;11:122. Epub 2012/10/09. doi: 10.1186/1475-2840-11-122. PubMed PMID: 23039216; PubMed Central PMCID: PMCPMC3508810.
17. Chen LK, Lin MH, Lai HY, Hwang SJ. Care of patients with diabetes mellitus in long-term care facilities in Taiwan: diagnosis, glycemic control, hypoglycemia, and functional status. J Am Geriatr Soc. 2008;56(10):1975-6. Epub 2008/12/05. doi: 10.1111/j.1532-5415.2008.01904.x. PubMed PMID: 19054212.
18. Freeman J. Management of hypoglycemia in older adults with type 2 diabetes. Postgrad Med. 2019;131(4):241-50. Epub 2019/02/07. doi: 10.1080/00325481.2019.1578590. PubMed PMID: 30724638.
19. Workgroup on Hypoglycemia ADA. Defining and reporting hypoglycemia in diabetes: a report from the American Diabetes Association Workgroup on Hypoglycemia. Diabetes Care. 2005;28(5):1245-9. Epub 2005/04/28. doi: 10.2337/diacare.28.5.1245. PubMed PMID: 15855602.
20. Seaquist ER, Anderson J, Childs B, Cryer P, Dagogo-Jack S, Fish L, et al. Hypoglycemia and diabetes: a report of a workgroup of the American Diabetes Association and the Endocrine Society. Diabetes Care. 2013;36(5):1384-95. Epub 2013/04/17. doi: 10.2337/dc12-2480. PubMed PMID: 23589542; PubMed Central PMCID: PMCPMC3631867.
21. International Hypoglycaemia Study G. Glucose Concentrations of Less Than 3.0 mmol/L (54 mg/dL) Should Be Reported in Clinical Trials: A Joint Position Statement of the American Diabetes Association and the European Association for the Study of Diabetes. Diabetes Care. 2017;40(1):155-7. Epub 2016/11/23. doi: 10.2337/dc16-2215. PubMed PMID: 27872155.
22. Charlson M, Szatrowski TP, Peterson J, Gold J. Validation of a combined comorbidity index. J Clin Epidemiol. 1994;47(11):1245-51. Epub 1994/11/01. doi: 10.1016/0895-4356(94)90129-5. PubMed PMID: 7722560.
23. Charan J, Biswas T. How to calculate sample size for different study designs in medical research? Indian J Psychol Med. 2013;35(2):121-6. Epub 2013/09/21. doi: 10.4103/0253-7176.116232. PubMed PMID: 24049221; PubMed Central PMCID: PMCPMC3775042.
24. Miller CD, Phillips LS, Ziemer DC, Gallina DL, Cook CB, El-Kebbi IM. Hypoglycemia in patients with type 2 diabetes mellitus. Arch Intern Med. 2001;161(13):1653-9. Epub 2001/07/24. doi: 10.1001/archinte.161.13.1653. PubMed PMID: 11434798.
25. Edridge CL, Dunkley AJ, Bodicoat DH, Rose TC, Gray LJ, Davies MJ, et al. Prevalence and Incidence of Hypoglycaemia in 532,542 People with Type 2 Diabetes on Oral Therapies and Insulin: A Systematic Review and Meta-Analysis of Population Based Studies. PLoS One. 2015;10(6):e0126427. Epub 2015/06/11. doi: 10.1371/journal.pone.0126427. PubMed PMID: 26061690; PubMed Central PMCID: PMCPMC4465495 Nordisk, Sanofi-Aventis, Lilly and Merck Sharp & Dohme. He has received grants in support of investigator and investigator initiated trials from Novartis, Novo Nordisk, Sanofi-Aventis, Lilly, Pfizer, Boehringer Ingelheim and Merck Sharp & Dohme. MJD has acted as consultant, advisory board member and speaker for Novartis, Novo Nordisk, Sanofi-Aventis, Lilly, Merck Sharp & Dohme, Boehringer Ingelheim and Roche. She has received grants in support of investigator and investigator initiated trials from Novartis, Novo Nordisk, Sanofi-Aventis, Lilly, Pfizer, Merck Sharp & Dohme and GlaxoSmithKline. AJD has received sponsorship for attending conferences.
26. Samya V, Shriraam V, Jasmine A, Akila GV, Anitha Rani M, Durai V, et al. Prevalence of Hypoglycemia Among Patients With Type 2 Diabetes Mellitus in a Rural Health Center in South India. J Prim Care Community Health. 2019;10:2150132719880638. Epub 2019/10/22. doi: 10.1177/2150132719880638. PubMed PMID: 31631765; PubMed Central PMCID: PMCPMC6804359.

27. Chow E, Bernjak A, Williams S, Fawdry RA, Hibbert S, Freeman J, et al. Risk of cardiac arrhythmias during hypoglycemia in patients with type 2 diabetes and cardiovascular risk. Diabetes. 2014;63(5):1738-47. Epub 2014/04/24. doi: 10.2337/db13-0468. PubMed PMID: 24757202.
28. Moffet HH, Huang ES, Liu JY, Parker MM, Lipska KJ, Laiteerapong N, et al. Severe hypoglycemia and falls in older adults with diabetes: The Diabetes & Aging Study. Diabet Epidemiol Manag. 2023;12. Epub 2023/11/03. doi: 10.1016/j.deman.2023.100162. PubMed PMID: 37920602; PubMed Central PMCID: PMCPMC10621321.
29. Naser AY, Wong ICK, Whittlesea C, Beykloo MY, Man KKC, Lau WCY, et al. Use of multiple antidiabetic medications in patients with diabetes and its association with hypoglycaemic events: a case-crossover study in Jordan. BMJ Open. 2018;8(11):e024909. Epub 2018/11/24. doi: 10.1136/bmjopen-2018-024909. PubMed PMID: 30467136; PubMed Central PMCID: PMCPMC6252777.
30. Seltzer HS. Drug-induced hypoglycemia. A review of 1418 cases. Endocrinol Metab Clin North Am. 1989;18(1):163-83. Epub 1989/03/01. PubMed PMID: 2645125.
31. Shorr RI, Ray WA, Daugherty JR, Griffin MR. Incidence and risk factors for serious hypoglycemia in older persons using insulin or sulfonylureas. Arch Intern Med. 1997;157(15):1681-6. Epub 1997/08/11. PubMed PMID: 9250229.
32. Yu S, Fu AZ, Engel SS, Shankar RR, Radican L. Association between hypoglycemia risk and hemoglobin A1C in patients with type 2 diabetes mellitus. Curr Med Res Opin. 2016;32(8):1409-16. Epub 2016/04/08. doi: 10.1080/03007995.2016.1176017. PubMed PMID: 27053004.
33. Zhao MJY, Prentice JC, Mohr DC, Conlin PR. Association between hemoglobin A1c variability and hypoglycemia-related hospitalizations in veterans with diabetes mellitus. BMJ Open Diabetes Res Care. 2021;9(1). Epub 2021/01/13. doi: 10.1136/bmjdrc-2020-001797. PubMed PMID: 33431600; PubMed Central PMCID: PMCPMC7802724.
34. American Diabetes Association Professional Practice C. 10. Cardiovascular Disease and Risk Management: Standards of Medical Care in Diabetes-2022. Diabetes Care. 2022;45(Suppl 1):S144-S74. Epub 2021/12/30. doi: 10.2337/dc22-S010. PubMed PMID: 34964815.
35. ElSayed NA, Aleppo G, Aroda VR, Bannuru RR, Brown FM, Bruemmer D, et al. 2. Classification and Diagnosis of Diabetes: Standards of Care in Diabetes-2023. Diabetes Care. 2023;46(Suppl 1):S19-S40. Epub 2022/12/13. doi: 10.2337/dc23-S002. PubMed PMID: 36507649; PubMed Central PMCID: PMCPMC9810477.
36. de Boer IH, Bangalore S, Benetos A, Davis AM, Michos ED, Muntner P, et al. Diabetes and Hypertension: A Position Statement by the American Diabetes Association. Diabetes Care. 2017;40(9):1273-84. Epub 2017/08/24. doi: 10.2337/dci17-0026. PubMed PMID: 28830958.
37. Dungan K, Merrill J, Long C, Binkley P. Effect of beta blocker use and type on hypoglycemia risk among hospitalized insulin requiring patients. Cardiovasc Diabetol. 2019;18(1):163. Epub 2019/11/30. doi: 10.1186/s12933-019-0967-1. PubMed PMID: 31775749; PubMed Central PMCID: PMCPMC6882013.
38. Moen MF, Zhan M, Hsu VD, Walker LD, Einhorn LM, Seliger SL, et al. Frequency of hypoglycemia and its significance in chronic kidney disease. Clin J Am Soc Nephrol. 2009;4(6):1121-7. Epub 2009/05/09. doi: 10.2215/CJN.00800209. PubMed PMID: 19423569; PubMed Central PMCID: PMCPMC2689888.
39. Kalra S, Unnikrishnan AG, Sahay R. The hypoglycemic side of hypothyroidism. Indian J Endocrinol Metab. 2014;18(1):1-3. Epub 2014/04/05. doi: 10.4103/2230-8210.126517. PubMed PMID: 24701422; PubMed Central PMCID: PMCPMC3968713.
40. Anno T, Kaneto H, Shigemoto R, Kawasaki F, Kawai Y, Urata N, et al. Hypoinsulinemic hypoglycemia triggered by liver injury in elderly subjects with low body weight: case reports. Endocrinol Diabetes Metab Case Rep. 2018;2018. Epub 2018/03/03. doi: 10.1530/EDM-17-0155. PubMed PMID: 29497536; PubMed Central PMCID: PMCPMC5825835.
41. Ensling M, Steinmann W, Whaley-Connell A. Hypoglycemia: A Possible Link between Insulin Resistance, Metabolic Dyslipidemia, and Heart and Kidney Disease (the Cardiorenal Syndrome).

Cardiorenal Med. 2011;1(1):67-74. Epub 2012/01/20. doi: 10.1159/000322886. PubMed PMID: 22258467; PubMed Central PMCID: PMCPMC3101522.
42. Khanimov I, Segal G, Wainstein J, Boaz M, Shimonov M, Leibovitz E. High-Intensity Statins Are Associated With Increased Incidence of Hypoglycemia During Hospitalization of Individuals Not Critically Ill. Am J Med. 2019;132(11):1305-10. Epub 2019/06/25. doi: 10.1016/j.amjmed.2019.04.050. PubMed PMID: 31233703.
43. Nozue T, Michishita I, Mizuguchi I. Effects of ezetimibe on glucose metabolism in patients with type 2 diabetes: A 12-week, open-label, uncontrolled, pilot study. Curr Ther Res Clin Exp. 2010;71(4):252-8. Epub 2010/08/01. doi: 10.1016/j.curtheres.2010.08.002. PubMed PMID: 24688147; PubMed Central PMCID: PMCPMC3969592.
44. Betten DP, Castle DJ, Hughes MJ, Henney JN. Frequency of return visits to the emergency department in patients discharged following hypoglycemia episodes. Int J Emerg Med. 2018;11(1):28. Epub 2018/05/26. doi: 10.1186/s12245-018-0186-7. PubMed PMID: 29799604; PubMed Central PMCID: PMCPMC5968011.

Figure Click here to access/download;Figure;Tables last Updated.docx

Table 1

Demographic characteristics of the study patients

**Variable**		**Total population (n=640)**			
		Mean (±SD) or n (%)[a]	Hypoglycemia episodes** (n= 139) 21.7% Mean (±SD) or n (%)[a]	No hypoglycemia episodes** (n= 501) 78.3% Mean (±SD) or n (%)[a]	P-value
**Age**		67.19 (±5.69)	66.74 (±5.51)	67.31 (±5.73)	0.296
**Gender**	Male	283 (44.22)	48 (16.96)	235 (83.04)	
	Female	357 (55.78)	91 (25.49)	266 (74.51)	0.009
**Body mass index (kg/m2)**	Normal	112 (17.50)	29 (25.89)	83 (74.11)	0.621
	Overweight	233 (36.40)	49 (21.03)	184 (78.97)	
	Obese	295 (46.10)	62 (21.02)	233 (78.98)	
**Marital status**	Married	530 (82.81)	115 (21.70)	415 (78.30)	0.182
	Single/other	110 (17.19)	32 (29.10)	78 (70.90)	
**Living conditions**	Not living alone	538 (84.10)	122 (22.68)	416 (77.32)	0.586
	Living alone	102 (15.90)	42 (41.18)	60 (58.82)	
**Residency**	City	311 (48.59)	61 (19.61)	250 (80.39)	0.225
	Countryside	329 (51.41)	78 (20.53)	302 (79.47)	
**Educational level**	College/university	260 (40.63)	61 (23.46)	199 (76.54)	0.376
	Less than college	380 (59.38)	56 (17.50)	264 (82.50)	
**Job Status**	Retired/Unemployed	539 (84.22)	127 (23.56)	412 (76.44)	0.172
	Employed	101 (15.78)	38 (37.62)	63 (62.38)	

**Monthly income**	More than 1000 JD*	147 (22.97)	41 (27.89)	106 (72.11)	0.110
	500-1000 JD*	158 (24.69)	33 (20.89)	125 (79.11)	
	Less than 500 JD*	335 (52.34)	65 (19.40)	270 (80.60)	
**Smoking status**		161 (25.16)	38 (23.60)	123 (76.40)	0.503
**Physical activities**		186 (29.06)	44 (23.66)	142 (76.34)	0.447
**Family history of Diabetes Miletus**		358 (55.94)	93 (25.98)	265 (74.02)	0.003

*Jordanian dinar; SD: Standard deviation; **hypoglycemia within the previous three months.

Figure 1: Patients distribution according to hypoglycemia severity levels (n=139)

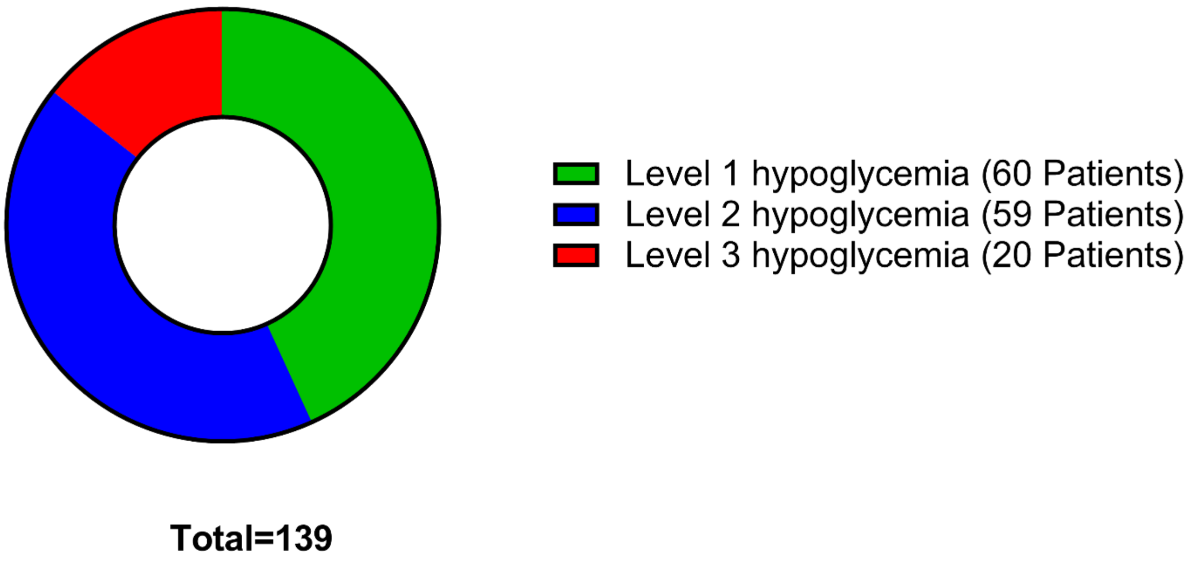

Table 2
Disease characteristics of the study patients

**Variables**	**Total population (n=640)**			
	**Mean (±SD) or n (%)[a]**	**Hypoglycemia episodes [1] (n= 139) Mean (±SD) or n (%)[a]**	**No hypoglycemia episodes [1] (n= 501) Mean (±SD) or n (%)[a]**	**P-value**
**Number of other chronic diseases**	2.72 (±1.47)	2.60 (±1.38)	2.76 (±1.49)	0.273
**Hypertension**	507 (79.22)	108 (21.30)	399 (78.70)	0.617
**Dyslipidemia**	372 (58.13)	90 (24.19)	282 (75.81)	0.074
**Chronic kidney disease**	55 (8.59)	17 (30.91)	38 (69.09)	0.084
**Chronic obstructive pulmonary disease**	16 (2.50)	3 (18.75)	13 (81.25)	0.771
**Leukemia**	3 (0.47)	0 (0.00)	3 (100.00)	0.360
**Lymphoma**	3 (0.47)	1 (33.33)	2 (66.67)	0.625
**Stroke**	49 (7.66)	11 (22.45)	38 (77.55)	0.897
**Myocardium infarction**	103 (16.09)	19 (18.45)	84 (81.55)	0.379
**Heart failure**	48 (7.50)	10 (20.83)	38 (79.17)	0.877
**Peptic ulcer disease**	9 (1.41)	2 (22.22)	7 (77.78)	0.950
**Cancer****	33 (5.16)	9 (27.27)	24 (72.73)	0.427
**Liver disease**	8 (1.25)	4 (50.00)	4 (50.00)	0.051
**Thyroid dysfunction**	55 (8.59)	11 (20.00)	44 (80.00)	0.746
**Asthma**	37 (5.79)	9 (24.32)	28 (75.68)	0.696
**Charlson comorbidity index[c]**	3.83 (±0.91)	3.77 (±0.85)	3.84 (±0.93)	0.408

[1]Hypoglycemia within the previous three months; [a] Standard deviation ;*P-value for variables significantly associated with hypoglycemia;**Solid tumor.

[c] Charlson comorbidity index calculated to assess the severity of comorbid diseases.

Table 3

Biomedical variables of the study patients

Variable	Total population (n=640)			
	Mean (±SD)*	Hypoglycemia episodes (n= 139) Mean (±SD)*	No hypoglycemia episodes (n=501) Mean (±SD)*	P-value
**HBA1C (%)**	7.70 (±1.68)	7.72 (±1.40)	7.70 (±1.74)	0.940
**Random blood glucose (mmol/L)** [a]	10.64 (±12.95)	9.91 (±5.09)	10.80 (±14.04)	0.742
**Fasting blood glucose (mmol/L)**[b]	10.16 (±20.20)	14.66 (±46.70)	9.11 (±3.64)	0.061
**Hypoglycemia value (mg/dl)**[c]	56.46 (±11.44)			
**Total Cholesterol (mmol/L)**[d]	6.22 (±26.20)	4.24 (1.21)	6.69 (29.14)	0.451
**Low-density lipoprotein (mmol/L)**[e]	2.99 (±7.99)	2.31 (0.89)	3.15 (±8.87)	0.403
**High-density lipoprotein (mmol/L)**[f]	1.63 (±5.74)	1.17 (±0.50)	1.74 (±6.37)	0.432
**Triglyceride (mmol/L)**[g]	2.76 (±10.47)	1.97 (±0.82)	2.95 (±11.65)	0.453
**Systolic blood pressure (mmHg)**	136.05 (±21.35)	140.07 (±26.12)	135.09 (±19.98)	0.052
**Diastolic blood pressure (mmHg)**	79.17 (±10.51)	80.03 (±12.10)	78.97 (±10.10)	0.399

*Standard deviation;[1]Variables significantly associated with hypoglycemia.

[a,b,c,d,e,f,g] The normal ranges for each variable, respectively, are: [a] Less than 11.1 mmol/L; (3.9-5.5mmol/L); [b] value of hypoglycemia considered when blood glucose is ≤70mg/dL; [c] Less than 5.2 mmol/L; [d] Less than 3.4 mmol/L; [e] Less than 2.6 mmol/L; [f] Greater than 1.3 mmol/L for females and greater than 1.0 mmol/L for males; [g] Less than 1.7 mmol/L.

Table 4

Medication characteristics of the patients and outcomes of the three months follow up period.

**Variables**		**Total population (n=640)**			
		**Mean (±SD) or n (%)***	**Hypoglycemia episodes (n= 139) Mean (±SD) or n (%)***	**No hypoglycemia episodes (n= 501) Mean (±SD) or n (%)***	**P-value**
**Diabetes medications frequency**			2.19 (±0.67)	2.22 (±0.64)	0.660
	Once	77 (12.03)	19 (24.68)	58 (75.32)	0.870
	Twice	351 (54.84)	75 (21.37)	276 (78.63)	
	Three times and more	212 (33.13)	45 (21.05)	167 (78.95)	
**Number of diabetes medications[1]**		1.70 (±0.87)	1.78 (±0.94)	1.68 (±0.85)	0.215
**Number of total medications**		6.59 (±2.72)	6.16 (±2.44)	6.71 (±2.78)	0.034
**Hospital admissions[b,1]**		115 (17.97)	24 (27.59)	63 (72.41)	0.020
**Emergency department visits[c]**		52 (8.13)	12 (25.53)	35 (74.47)	0.390
**Hospital admissions and emergency department visits[1]**		112 (22.40)	30 (26.79)	82 (73.21)	0.021

*Standard deviation; [a] hypoglycemia: considered when blood glucose is ≤70mg/dl.

[1] Variables significantly associated with hypoglycemia.

[b] Hospital admissions reasons during three months follow up were (asthma exacerbations, hypoglycemia, acute kidney injury, acute coronary syndrome, low oxygen saturation, toxic thyroiditis, hypoglycemia, diabetic foot infection, heart failure exacerbation, intracranial hemorrhage , stroke , esophagitis , pulmonary edema , pulmonary fibrosis).

[c]Emergency department visits reasons (Allergy, hypoglycemia, anemia, drowsiness, COVID19, fatigue, flu, hypertensive crises, hemorrhoids, shortness of breath, tachycardia, urinary tract infections , chest pain , diarrhea , peripheral edema , epigastric pain , hyperglycemia and severe headache).

Table 5
Association between specific antidiabetic medication and hypoglycemia occurrence.

Medication Category	N (%)	Hypoglycemia episodes	No Hypoglycemia episodes	P-value
**Biguanides (Metformin)**	501 (78.28)	107 (21.36)	394 (78.64)	0.674
**Sulfonylurea**	264 (41.25)	54 (20.45)	210 (79.55)	0.516
**Insulin**	243 (37.97)	65 (26.75)	178 (73.25)	0.016
**DPP4-I**	116 (18.13)	31 (26.72)	85 (73.28)	0.148
**GLP-1A**	3 (0.47)	1 (33.33)	2 (66.67)	0.625
**SGLT2-I**	40 (6.25)	10 (25.00)	30 (75.00)	0.603

DPP4-I: Dipeptidyl peptidase-4 inhibitor, GLP-1A Glucagon-Like Peptide-1 Receptor Agonists, SGLT2-I: Sodium-Glucose Transport Protein 2 (SGLT2) Inhibitors

Table 6
Predictors of hypoglycemia in elderly patients with type 2 DM.

Variable	OR[a]	95% CI [b]	P-value
**Number of Diabetes medications**	1.28	0.96-1.70	0.094
**Gender (Female)**	1.51	0.83-2.73	0.176
**Marital status (Married)**	1.63	0.87-3.06	0.126
**Liver disease ****	7.43	1.38-40.15	0.020
**Physical activities**	1.67	0.95-2.91	0.073
**Dyslipidemia****	1.87	1.06-3.30	0.032
**Job status (Employed)**	0.37	0.08-1.66	0.193
**Insulin ****	1.88	1.13-3.13	0.015
**Family history of Diabetes Mellitus (with)****	1.82	1.08-3.05	0.023
**Smoking (Smokers)**	1.77	0.90-3.46	0.097

**variables significantly associated with hypoglycemia; [a] odds ratio; [b] confidence interval.

Table 7
Variables associated with hospital admissions and emergency department visits*

Variable	OR[a]	(95% CI)[b]	P-value
Hypoglycemia**	1.95	(1.25-3.04)	0.003
Gender (Female)	0.69	(0.45-1.06)	0.090
Marital status (Married)	1.56	(0.91-2.65)	0.104
Education (college/university)**	0.61	(0.40-0.94)	0.026
Family history of DM (with)	1.23	(0.82-1.85)	0.309
Number of Diabetes medications	0.87	(0.69-1.10)	0.259
CCI	1.09	(0.88-1.35)	0.409

*The independent variables included in the regression model; ** variables significantly associated with hospital admissions and emergency department visits; [a] odd ratio; [b] confidence interval; [c] diabetes mellitus.

5. Review Comments to the Author

Please use the space provided to explain your answers to the questions above. You may also include additional comments for the author, including concerns about dual publication, research ethics, or publication ethics. (Please upload your review as an attachment if it exceeds 20,000 characters)

Reviewer #1: Dear Authors,
This manuscript addresses a significant subject due to the relationship of hypoglycemia with morbidity and mortality, especially in the elderly population, and the unawareness of such complications and its predisposing factors among individuals and their caregivers. However, some reviews are necessary.

The authors evaluated the prevalence in three months and compared it with the prevalence reported in a study that evaluated episodes per patient per year.

The paper (Ref 25) had estimated both prevalence and incidence rates. In the current study, we have compared the prevalence measures only.

It would be interesting to describe the severity of hypoglycemia.

Severity of hypoglycemia was assessed and described in figure (1), a new added figure

They also explain that they calculated the sample. However, in the item 'sample size calculation," it says: "385 subjects was required for achieving a 95% confidence interval with a margin of error of 5%", and later in the 'Study limitations' - "A relatively small number of elderly diabetic?? patients were evaluated in this study". The authors could describe how they calculated the sample.

Since the authors have increased the sample size to include (640 vs 500 patients), we believe that sample is more representative and exceeds the minim required number by around 2 folds. We have removed the limitation sentence "A relatively small number of elderly diabetic?? patients were evaluated in this study" to avoid any confusion to the readers.

In the results, the authors reported that "93.78% were not living alone, 59.84% were not living alone", which is confounding.

All typos and grammatical mistakes were corrected in this manuscript.

In Table 3, an item is apparently missing below 'Emergency Department visits', and it has a significant result.

The item is: Having either hospital admission or emergency visit

Moreover, it would be interesting to include the treatment used for diabetes by the participants.
Treatment used was described in table #5 (a newly added table)

In Table 4, HbA1C, hypoglycemia value and hemoglobin are not in the SI units.

Biolabs values are presented in table 3 now. The lab results were reported according to the SI units. Hemoglobin values were removed to include only most relevant lab parameters to their DM conditions.

Besides, the manuscript deserves a writing review due to mistakes in grammar, punctuation, and abbreviations, and it sometimes took effort to understand.
Yours Sincerely

All typos and grammatical mistakes were corrected in this manuscript. Many parts have been rewritten to reflect the new sample size results…etc.

Reviewer #2: The current study assesses the "prevalence, predictors, and factors associated with hypoglycemia events in geriatric patients with type 2 diabetes mellitus." The manuscript is well written and provides important information regarding the management of diabetes in patients. However, many reports/articles have been conducted/produced on the topic, thus authors should clearly indicate what is the uniqueness or the value of the current report.

The prevalence and predictors of hypoglycemia were not evaluated in Jordan and very few studies were conducted in the middle east region.
Minor comments
Its better to define type 2 diabetes mellitus (DM) very early/at first mention. In fact, abbreviations should be used checked and consistently throughout the manuscript

We did that, please view the manuscript.

Do you have a reference for "Sample size calculation"?

The sample size was calculated based on formula presented in the following paper (ref#23) Charan J, Biswas T. How to calculate sample size for different study designs in medical research? Indian J Psychol Med. 2013;35(2):121-6. Epub 2013/09/21. doi: 10.4103/0253-7176.116232. PubMed PMID: 24049221; PubMed Central PMCID: PMCPMC3775042.

Does type of medication influence the rate of hypoglycemia in patients? Authors should clearly describe this within the manuscript

Medications prescribed were clearly presented in table #5

What type of liver disease is being discussed?
Liver diseases included fatty liver disease, cirrhosis, hepatitis B.

How does the percentage achieved on the number of cases compare with other that seen in other world regions.
We have included a number of previous reports to compare our hypoglycemia prevalnce to their results (please view the discussion part)

Does diet and lifestyle impact hypoglycemia, especially exercise. This should be clearly discussed within the manuscript
Non-significant association (P value 0.152)
