# Supplementary material for: Prevalence and predictors of hypoglycemia in older outpatients with type 2 diabetes mellitus
Source: PLoS One. 2024 Aug 29;19(8):e0309618. doi: 10.1371/journal.pone.0309618 (PMC11361436; doi:10.1371/journal.pone.0309618)
Supplement: Attachment — Submitted filename: Response.docx [file pone.0309618.s002.docx]

5. Review Comments to the Author

Please use the space provided to explain your answers to the questions above. You may also include additional comments for the author, including concerns about dual publication, research ethics, or publication ethics. (Please upload your review as an attachment if it exceeds 20,000 characters)

**Reviewer #1: Dear Authors,
This manuscript addresses a significant subject due to the relationship of hypoglycemia with morbidity and mortality, especially in the elderly population, and the unawareness of such complications and its predisposing factors among individuals and their caregivers. However, some reviews are necessary.**

The authors evaluated the prevalence in three months and compared it with the prevalence reported in a study that evaluated episodes per patient per year.

The paper (Ref 25) had estimated both prevalence and incidence rates. In the current study, we have compared the prevalence measures only.

It would be interesting to describe the severity of hypoglycemia.

Severity of hypoglycemia was assessed and described in figure (1), a new added figure

They also explain that they calculated the sample. However, in the item 'sample size calculation," it says: "385 subjects was required for achieving a 95% confidence interval with a margin of error of 5%", and later in the 'Study limitations' - "A relatively small number of elderly diabetic?? patients were evaluated in this study". The authors could describe how they calculated the sample.

Since the authors have increased the sample size to include (640 vs 500 patients), we believe that sample is more representative and exceeds the minim required number by around 2 folds. We have removed the limitation sentence "A relatively small number of elderly diabetic?? patients were evaluated in this study" to avoid any confusion to the readers.

In the results, the authors reported that "93.78% were not living alone, 59.84% were not living alone", which is confounding.

All typos and grammatical mistakes were corrected in this manuscript.

In Table 3, an item is apparently missing below 'Emergency Department visits', and it has a significant result.

The item is: Having either hospital admission or emergency visit

Moreover, it would be interesting to include the treatment used for diabetes by the participants.

Treatment used was described in table #5 (a newly added table)

In Table 4, HbA1C, hypoglycemia value and hemoglobin are not in the SI units.

Biolabs values are presented in table 3 now. The lab results were reported according to the SI units. Hemoglobin values were removed to include only most relevant lab parameters to their DM conditions.

Besides, the manuscript deserves a writing review due to mistakes in grammar, punctuation, and abbreviations, and it sometimes took effort to understand.
Yours Sincerely

All typos and grammatical mistakes were corrected in this manuscript. Many parts have been rewritten to reflect the new sample size results…etc.

**Reviewer #2: The current study assesses the “prevalence, predictors, and factors associated with hypoglycemia events in geriatric patients with type 2 diabetes mellitus.” The manuscript is well written and provides important information regarding the management of diabetes in patients. However, many reports/articles have been conducted/produced on the topic, thus authors should clearly indicate what is the uniqueness or the value of the current report.**

The prevalence and predictors of hypoglycemia were not evaluated in Jordan and very few studies were conducted in the middle east region.
Minor comments
Its better to define type 2 diabetes mellitus (DM) very early/at first mention. In fact, abbreviations should be used checked and consistently throughout the manuscript

We did that, please view the manuscript.

Do you have a reference for “Sample size calculation”?

The sample size was calculated based on formula presented in the following paper (ref#23)

Charan J, Biswas T. How to calculate sample size for different study designs in medical research? Indian J Psychol Med. 2013;35(2):121-6. Epub 2013/09/21. doi: 10.4103/0253-7176.116232. PubMed PMID: 24049221; PubMed Central PMCID: PMCPMC3775042.

Does type of medication influence the rate of hypoglycemia in patients? Authors should clearly describe this within the manuscript

Medications prescribed were clearly presented in table #5

What type of liver disease is being discussed?

Liver diseases included fatty liver disease, cirrhosis, hepatitis B.

How does the percentage achieved on the number of cases compare with other that seen in other world regions.

We have included a number of previous reports to compare our hypoglycemia prevalnce to their results (please view the discussion part )

Does diet and lifestyle impact hypoglycemia, especially exercise. This should be clearly discussed within the manuscript

Non-significant association (P value 0.152)
