# Supplementary material for: Prevalence and predictors of hypoglycemia in older outpatients with type 2 diabetes mellitus
Source: PLoS One. 2024 Aug 29;19(8):e0309618. doi: 10.1371/journal.pone.0309618 (PMC11361436; doi:10.1371/journal.pone.0309618)
Supplement: Attachment — Submitted filename: Response Letter to the Reviewers Comments.docx [file pone.0309618.s004.docx]

Response letter to the Reviewers’ Comments.

Reviewer #1: My comments are in the attached file. The sample size calculation should be clearly stated. The standard deviation for the mean total cholesterol and LDL-c is quite large. The author need to look at the data again. Discussion need to be re-done.

Would like to thank the reviewer for his/her valid comments. All comments were addressed in the main manuscript. Actually, the authors had rewritten the most part of the manuscript including introduction, methodology..etc.

Sample size calculation formula and details were addressed in the methodology part.

The authors have removed table number 4 which includes the biomedical data because of the issue of missing data and since we believe that it will not affect the essence and main conclusion of the manuscript.

“Cardiovascular diseases, dyslipidemia, hypothyroidism, and chronic kidney disease were the most common chronic conditions encountered in this study These were not stated in the results. There is no thyroid function test result or frequency of hypothyroidism. There is nothing about eGFR so CKD cannot be discussed.”

These comorbidities do co-exist among the study participants and they have been diagnosed with these conditions already. Please view table number 2 to view the frequency of these conditions.

Reviewer#2: 

1. The authors have mentioned that it is a cross sectional study. But they also say, they followed up OP patients for 3 months. Will this not come under a prospective cohort study? Thus, the hypoglycemia that they mention will be incidence and not prevalence.

Would like to thank the reviewer for his/her concern.

For the primary objective of measuring the prevalence and factors associated with hypoglycemia, a Cross-sectional study was conducted at King Abdullah University Hospital (KAUH) at the outpatient diabetes clinic over 10 months period, from October 1^st^, 2022 to August 1^st^, 2023. Over a three-month follow-up period, electronic medical records were utilized to prospectively track recruited patients for hospital admissions, emergency department visits, and mortality within the framework of a prospective cohort study design.

This issue has been clarified in the text.

2. There is no clear mention of when the electronic data of the patients were assessed – was it the baseline data at the first visit or was it during the admission later. Were the patient’s who were interviewed initially and that agreed to study were followed up for next 3 months or other patients who did not agree to participate in the study but were admitted for hypoglycemia were included?? Not clear from the manuscript.

Electronic data were assessed, and patients were interviewed at their first visit to the clinic. The same patients interviewed were followed up for the next 3 months for any emergency/hospital visits.

This issue has been clarified in the text.

3. In the limitations – the authors have mentioned about recall bias; it is unclear why recall bias when the source is electronic data.

First of All, apologize for any misunderstanding. We are here talking about hypoglycemia occurrences (if it happened for example, how many times, and blood glucose reported at that time). This question was answered through interviewing the patients and this might lead to a recall bias which could be associated with self-reporting.

This issue has been clarified in the text.

4. The sample size calculation – the basis needs to be explained.

Please view the methodology section for clarification.

5. When we say hypoglycemia prevalence among geriatric population with type 2 diabetes mellitus, it should include all elders with diabetes. When we include a hospital-based study among patients attending OP or IP for one or other health issues, it might not be representative of all elders with diabetes.

Agree with the reviewer on this. We have changed the title and any relevant data to focus on outpatient older patients type 2 DM patients.

Our future work would focus on the inpatient older subjects suffering from type 2 DM.

6. Further, in this study for prevalence of hypoglycemia, they are considering only patients who are getting admitted, visit emergency, or die in the next 3 months period. Will it not include only the severe cases and leave the milder cases of hypoglycemia? Can we call as prevalence of severe hypoglycemia among elderly withdiabetes?

Hypoglycemia was assessed during the interview (self-report) in the initial clinic visits. Readmissions and ED visits assessed were all-cause visits occurred during the three months follow up period. So we did not follow patients particularly for hypoglycemia. The main objective of this study was to assess the prevalence of hypoglycemia. As a secondary objective, we have evaluated the association between hypoglycemia (self-reported during the clinic visits), along with other potential predictors, and the occurrence of hospital admissions/emergency department visits during the follow up period.

7. In table 4, they mention hospital admissions as 115 and emergency visits as 52 and both as 112. Is it an arithmetic error, again not clear?

Patients how had hospital admissions **or** emergency department visits were 143. These numbers are actually number of patients not visits. Some patients had both, so it is expected to have the number of patients how had hospital admissions **or** emergency department visits to be lower than (115+52). The data are presented in **table 3**

8. In the tables 2- 4, it is given as hypoglycemia episodes, is it no of episodes or no of patients with hypoglycemia?

These are the number of patients who had at least one episode of hypoglycemia during the three months preceding the clinic visit.

9. In table 4, what is the denominator for the percentages calculated? It should be out of the no. of episodes. Here, it is neither no. of episodes nor no. of hospital admissions.

The denominator is the number of patients. For example: 501 patients were on Metformin (out of 640; 78.3%). 394 of them were among the group who did not report any hypoglycemic episode (out of 501 patients; 78.6%). 107 of them were among the group who did report at least one hypoglycemic episode (out of 139 patients; 76.98% [ corrected in the manuscript).

We apologize for mistakenly reporting row percentages rather than the column percentages. All percentages are now corrected, and column percentages are reported.
