# Supplementary material for: Prevalence and predictors of hypoglycemia in older outpatients with type 2 diabetes mellitus
Source: PLoS One. 2024 Aug 29;19(8):e0309618. doi: 10.1371/journal.pone.0309618 (PMC11361436; doi:10.1371/journal.pone.0309618)
Supplement: Attachment — Submitted filename: Hypoglycemia Comments.docx [file pone.0309618.s005.docx]

Response to Reviewers’ Comments to the Author

Reviewer #3: This article deals with an important issue - hypoglycemia in diabetes mellitus. The design, materials and methods, as well as the results, are classically structured. The examination reveals several points:

**1. Add the date and number of the ethics committee in the abstract:**

The date and number of the ethical committee decision was added as recommended.

**2. Add type of diabetes mellitus to the keywords**

Type 2 diabetes mellitus was added to the keywords part

**3. In the text also specify the number and date of the ethics committee minutes**

The date and number of the ethical committee decision was added as recommended under the methodology section.

**4. In the list of references, sources older than 5 years, where it is possible to review**

First, we would like to thank the reviewer for her valuable feedback on our manuscript. We appreciate your attention to the references included in our study.

1. References Highlighting Original Reports: We have ensured that the references include those that first reported significant findings related to our topic. These original studies are crucial as they lay the groundwork for subsequent research and provide the foundational context for our study. We have marked these original studies in the reference list to highlight their importance. (REF#: 12, 27, 29, and 33)
2. Comprehensive Inclusion of Prevalence Studies: In addition to the original reports, we have included a range of studies that discuss the prevalence of hypoglycemia. Our intention was to provide a comprehensive overview of the existing literature, capturing the various findings and perspectives across different studies. We believe this approach offers a more holistic view of the research landscape. (REF#: 18)
3. Updating References: We have recently updated some references to ensure the most current and relevant studies are cited, reflecting the latest advancements and data in the field. In doing so, we removed older references that have been superseded by more recent work. This was done to maintain the accuracy and relevance of our literature review. Please view the reference list for more details

**5. In the table the designation of glycated haemoglobin HBA1C, preferably use the correct abbreviation HbA1c**

The glycosylated Hemoglobin HbA1c was replaced by the Glycated Hemoglobin (HbA1c).

**Reviewer #4: The paper ** Prevalence and predictors of hypoglycemia in older outpatients with type 2 diabetes mellitus**was reviewed. The results revealed the study identified a considerable prevalence of hypoglycemia among older patients with
type 2 diabetes, particularly, among those with concurrent liver diseases and dyslipidemia. However, there are some concerns necessary to be clarified.
The major concern is that why dyslipidemia is associated with hypoglycemia? The discussion should mention and provide some explanations about the results- treatments associated? Or some confounding factors such as the patients with dyslipidemia treated by insulin.**

The following paragraph was added to the discussion part:

“Various pharmacological studies have examined the effects of drugs prescribed for dyslipidemia on the blood glucose level, with findings indicating that the administration of statins and ezetimibe may induce notable hypoglycemia [32, 33]. Consequently, the hypoglycemic events in patients with co-existed dyslipidemia may be attributable to the pharmacological effects of the administered drugs. Furthermore, insulin resistance is associated with dyslipidemia, creating a reciprocal relationship in which each condition has the potential to exacerbate the other [34], thereby necessitating mainly the administration of higher insulin doses [35]. This increase in dosage may, in turn, lead to a higher frequency of hypoglycemic episodes.”
